# A randomized clinical trial of cavity liners after selective caries removal: one-year follow-up

**DOI:** 10.1590/1678-7757-2018-0700

**Published:** 2019-06-03

**Authors:** Tássia Carina Stafuzza, Luciana Lourenço Ribeiro Vitor, Daniela Rios, Thiago Cruvinel, Natalino Loureço, Vivien Thiemy Sakai, Maria Aparecida Andrade Moreira Machado, Thais Marchini Oliveira

**Affiliations:** 1 Universidade de São Paulo, Faculdade de Odontologia de Bauru, Departamento de Odontopediatria, Ortodontia e Saúde Coletiva, Bauru, São Paulo, Brasil.; 2 Universidade Federal de Alfenas, Faculdade de Odontologia, Departamento de Clínica e Cirurgia, Alfenas, Minas Gerais, Brasil.

**Keywords:** Dental caries, Dental cavity liners, Primary teeth, Pediatric Dentistry

## Abstract

Alternatives for the treatment of caries disease, such as minimally invasive approaches, have been developed in recent years.

**Objective:**

To carry out clinical and radiographic evaluations of three cavity liners after selective caries removal.

**Methodology:**

Thirty-six primary molars with deep occlusal caries lesions without pulp involvement (from children of both genders, aged between 5 and 8 years) were randomly divided into the following groups: calcium hydroxide cement (CHC) group; mineral trioxide aggregate (MTA) group and Portland cement with added zirconium oxide (PCZ) group. The following-up period was 6- and 12-month. The clinical and radiographic success rates were evaluated through chi-square test. The radiographic measurements were compared by ANOVA followed by Tukey's test (p<0.05).

**Results:**

Thirty-six patients were included, but thirty-four returned for 12-month follow-up. The overall success rate of the therapy for the three groups was 94.11% and no statistically significant differences occurred in the comparison among groups (p>0.05). Nineteen radiographs were selected to measure the dentin barrier thickness. The intragroup comparison presented a statistically significant increase of the dentin barrier for all groups, at 12-month follow-up. However, the MTA group showed increase of the dentin barrier, over time, 6- to 12-month follow-up. The intergroup comparison revealed no statistically significant differences (p>0.05).

**Conclusion:**

The clinical and radiographic data showed that all cavity liners provided effective treatment of primary teeth after selective caries removal.

## Introduction

The selective caries removal is a minimally invasive approach consisting of non-removal of the affected dentin followed by hermetic sealing of the cavity.^1^ ^–^ ^3^ This technique reduces the number and diversity of the bacteria, stops the caries process,^4^ ^,^ ^5^ and decreases the risk to pulp exposure,^4^ ^–^ ^7^ presenting clinical advantage over complete caries removal.^5^

For restorative interventions cavity lining has been recommended for a long time.^8^ Researches has shown that Calcium Hydroxide (CH) promotes a decrease in the number of cariogenic bacteria, favoring remineralization.^9^ However, a systematic review of incompletely excavated teeth found a tendency to fail in CH lining cavities despite the adequacy of this protective material for deep carious lesions.^6^ CH exhibits unfavorable effects such as internal resorption, degradation over time and defects in dentin barrier formation.^10^ Because of this, other insoluble materials as Mineral Trioxide Aggregate (MTA) and Portland Cement (PC) may be used as cavity lining materials in primary teeth.^11^ Both, MTA and PC, have similar clinical, biological and mechanical properties.^12^ ^,^ ^13^ There are few studies that use PC in human teeth, but this material presented satisfactory clinical and radiographic results.^12^ ^–^ ^15^

Current evidence indicates that less invasive strategies are effective for managing carious lesion,^8^, but the literature is scarce in pointing out the ideal liner material that should be used on the remaining dentin in the selective caries removal in primary teeth. Clear recommendations on which liner should be placed after the selective caries removal are still necessary.^11^ ^,^ ^16^ Thus, the objective of this study was to perform clinical and radiographic evaluations of three cavity liners after selective caries removal. The null hypothesis was that the three cavity liners showed no differences in the comparison.

## Methodology

### Study approval and design

This study was approved by the Institutional Review Board (protocol no. 45955515.2.0000.5417) and registered in the Brazilian Registry of Clinical Trials (ReBEC; protocol RBR-5SB8SB).

Children were recruited for the trial at local municipal schools and the treatments were performed at the Pediatric Dentistry Clinic of the University. Children and legal guardians were instructed about the research and gave written informed consent (parents/guardians) and informed assent (children) for participation in the trial. We provided all dental care required by the participants and efforts were made to achieve adherence by explaining the treatment importance. Each participant was enrolled in the study for approximately thirteen months (one month for treatment and twelve follow-up months). During the study, all assistance was given to the participants and, upon completion of the study, the children continued to attend the university dental clinic.

**Figure 1:**
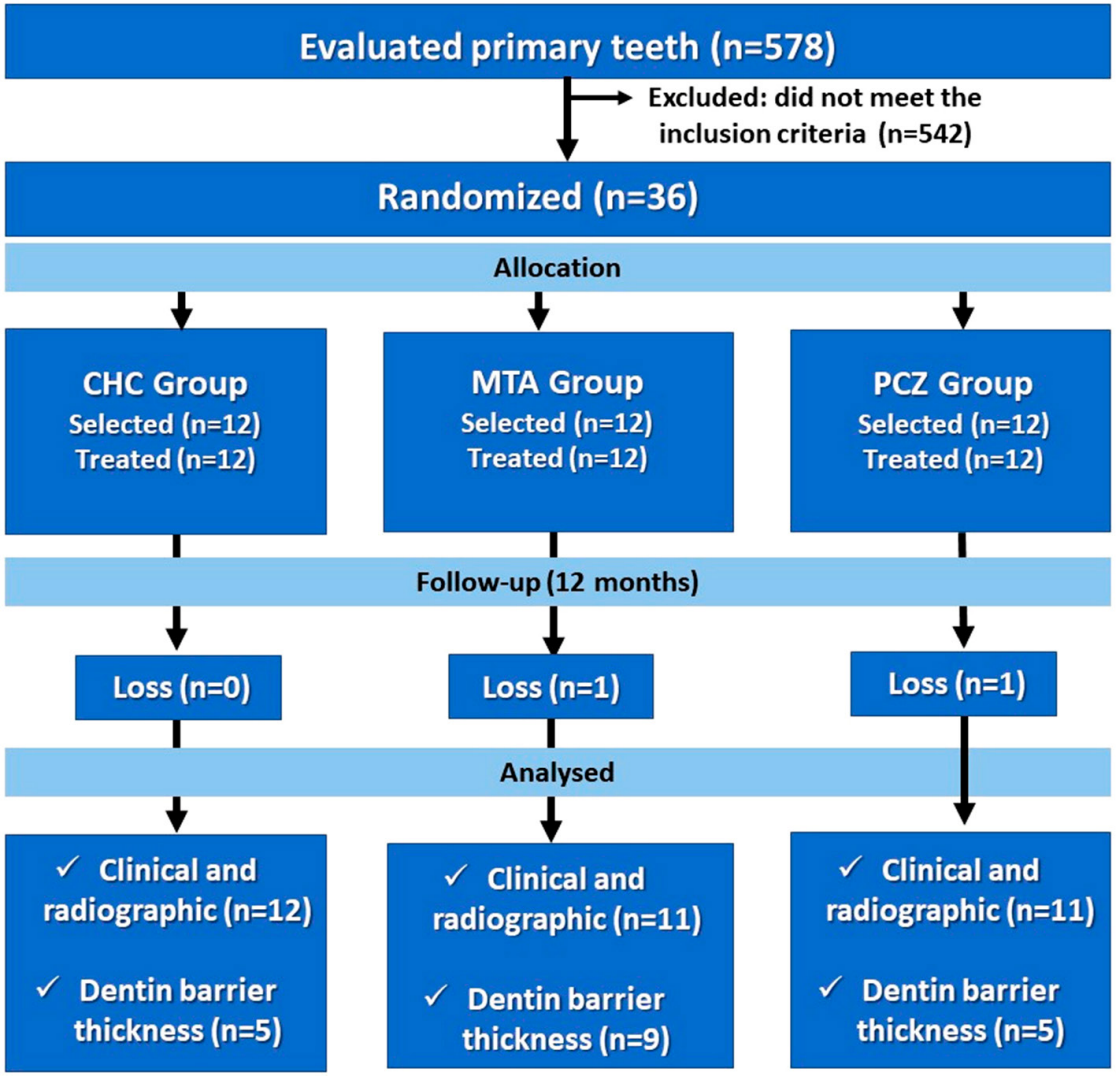
CONSORT study flowchart

This study was composed by three groups according to each material: Calcium Hydroxide Cement (Hydro C^®^/Dentsply Petrópolis, RJ, Brazil) group (CHC group); MTA (MTA Angelus^®^ Londrina, PR, Brazil) group (MTA group) and Portland Cement (Votorantim Cimentos São Paulo, SP, Brazil) added with zirconia oxide (Sigma-Aldrich Co., Saint Louis, USA) at 4:1 ratio for radiopacity^14^ group (PCZ group). The selected teeth were distributed through computerized stratified sampling (Microsoft Excel^®^) according to the child age in months and dmtf index. Each tooth was randomly allocated to receive one of the three cavity liners (Figure 1). The following-up period was 6- and 12-month.

### Sample selection

Inclusion criteria comprised children of both genders, aged between 5 and 8 years, with deep occlusal caries lesions (class I) without pulp involvement in first or second primary molars. Exclusion criteria were the presence of systemic disease, history of allergic reaction to dental materials, pulp exposure, painful symptomatology, dental mobility, abscess, furcation defects, periodontal diseases, and tooth with no restorative possibility.^13^ ^,^ ^14^

### Sample size

The minimum sample size calculation was performed based on data from a previous study.^17^ For the sample calculation, alpha and beta errors of 5% and 20% were considered respectively. It was estimated that 12 molars *per* group would be necessary to detect a difference between the materials. The final sample size was 36 molars.

### Clinical interventions

A previous pilot study was performed to ensure operator calibration. Detailed anamnesis and clinical examination were performed initially. To satisfy the inclusion and exclusion criteria a periapical radiograph of the eligible primary molar was taken. Radiographs were standardized using universal acrylic positioning stents.

At the second visit, after local anesthesia and rubber dam isolation, removal of enamel was performed using water-cooled diamond instruments.^9^ ^,^ ^14^ Caries was completely removed from the lateral walls with the aid of low speed round burs. Selective caries removal on pulpal wall was executed with the aid of manual excavators until firm dentin. CH and MTA were prepared according to the manufactures’ instructions on dental use and placed gently on the pulp wall with a thickness of approximately 1 mm. The same MTA powder/liquid ratio was used for PC manipulation. This material was also placed gently on the pulp wall until it reached 1 mm. Restoration was carried out with the use of resin-modified glass ionomer cement (Vitremer^®^ 3M/ESPE, São Paulo, SP, Brazil).^9^ ^,^ ^14^ If pulpal exposure occurred during excavation the tooth was excluded from the study and a pulpotomy was accomplished.

Clinical analyzes are based on a form and were performed by an operator who was blind to the clinical procedure. At following-up period, clinical criteria were evaluated: local pain, dental mobility, percussion sensitivity, periapical abscess, and restoration failure such as secondary caries.

### Radiographic procedures

Caution was taken regarding the risks related to the radiographic shots. During all radiographic shots, all children wore lead apron and thyroid collar. Radiographs were standardized using universal acrylic positioning stents and ultra-speed x-ray film size 1 (Kodak, Rochester, NY, USA). The focus/film distance was approximately 20 cm. All radiographs were obtained with a dental x-ray device at 70 kV and 10 mA, with exposure time of 0.5 seconds. The x-ray film processing procedures assured the image quality control.^9^

Radiographic analyzes are based on a form and were performed by a single calibrated operator who was blind to the clinical procedure. The evaluated radiographic criteria were furcation lesion, restoration failure, internal and external resorption.

The radiographs obtained at immediate post-operative period, at 6 and 12 month following-up period were subsequently digitized (EPSON PERFECTION V750 PRO Dual Lens System - High Pass Optics - Seiko Epson Corporation, Tokyo, Japan). Settings for scanning images included: positive color film; 16-bit gray scale and 300 ppp image resolution. The digitized images were transferred to a notebook ASUS X450C Series (ASUSTeK Computer Inc., Taiwan, China) and stored as .JPG files. All images were analyzed through Image J software (National Institutes of Health, NY, USA) 64-bit version, by a single trained and calibrated examiner.^18^ The dentin barrier thickness was verified by measuring the height of the pulp chamber.^18^ To ensure accurate measurement of reference points, each scanned image was measured more than once by one calibrated examiner with a 15-day interval between measurements. The measurements on the digitized images were performed by one investigator in this study who was blind to the clinical procedure. The measurements were obtained in the following radiographs: immediate postoperative, 6 and 12 months of follow-up.^19^ Variations in the dimensions of the pulp chamber due to different ages were not considered in this study.^20^

### Statistical analysis

All data were analyzed using the PASW Statistics 20 software (SPSS, Hong Kong, China). Intra-examiner reliability was verified by casual and systematic error.^21^ The clinical and radiographic success rates were evaluated through chi-square test. The radiographic measurements were compared by ANOVA followed by Tukey's test. A level of significance of 5% was adopted.

## Results

### Patient sample and study design

Of 578 evaluated molars, 542 did not fill the inclusion criteria and were excluded from the study. The sample was composed by thirty-six primary molars from twenty-eight children (thirteen boys and fifteen girls). The age ranged from 5 to 8 years with mean ± standard deviation of 76.8 ±1.578 months and mean *dmtf* index of 4.91 *per* group. No statistically significant differences occurred among groups in relation to age and *dmtf* index (p>0.05). The procedures performed in this study are unlikely to result in any adverse effects. Side effects are usually expected in any conventional dental treatment performed in pediatric dentistry practice. During treatment, there was no case of pulpal exposure.

### Clinical and radiographic assessment

The systematic and casual error was verified, and no statistically significant differences occurred (p>0.05). Thirty-six patients were included, but thirty-four returned for 12-month follow-up. The overall success rate of the therapy for the three groups was 94.11% and no statistically significant differences occurred in the comparison among groups (p>0.05) (Tables 1 and 2).

**Table 1:** Clinical assessment of the use of different cavity liners in the selective caries removal from primary teeth, at 12-month follow-up

Group (cavity liner)	Caries symptomatology (local pain)	Tooth mobility	Sensitivity to percussion	Presence of fistula/abscess	Restoration failure
CHC	0/12	0/12	0/12	0/12	0/12
MTA	0/11	0/11	0/11	0/11	0/11
PCZ	0/11	0/11	0/11	0/11	02/11
Total	0/34	0/34	0/34	0/34	02/34
	0%	0%	0%	0%	6%

Values represent the number of teeth treated, per group

**Table 2:** Radiographic assessment of the use of different cavity liners in the selective caries removal from primary teeth, at 12-month follow-up

Group (cavity liner)	Internal resorption	External resorption	Furcation lesion	Restoration
CHC	0/12	0/12	0/12	0/12
MTA	0/11	0/11	0/11	0/11
PCZ	0/11	0/11	0/11	02/11
Total	0/34	0/34	0/34	02/34
	0%	0%	0%	6%

Values represent the number of teeth treated, per group

Thirty-four radiographs were selected to measure the pulp chamber height. Fifteen radiographs of maxillary molars were excluded due to the superposition of the roots. The intragroup comparison presented a statistically significant increase of the dentin barrier for all groups, at 12-month follow-up. However, the MTA group showed increase of the dentin barrier, over time, 6- to 12-month follow-up (Table 3). The intergroup comparison revealed no statistically significant differences (p>0.05).

**Table 3:** Intragroup comparisons of the pulp chamber height (ANOVA followed by Tukey's test, p<0.05)

	Height of the pulp chamber (mean±SD, in mm)			
Group	Immediate post-operative	6-months control	12-months control	p
CHC	1.187±0.394^a^	1.00±0.474^ab^	0.812±0.338^b^	0.0317*
MTA	0.872±0.184^a^	0.767±0.188b	0.650±0.156^c^	<0.0001*
PCZ	0.965±0.333^a^	0.842±0.342^ab^	0.708±0.213^b^	0.0052*

* Statistically significant difference (p<0.05)

Different lowercase letters indicate statistically significant differences (intragroup comparison)

## Discussion

The present randomized clinical study applied a standard methodology well described in the literature^11^ ^,^ ^17^ ^,^ ^22^ ^,^ ^23^ to evaluate the *in vivo* response of different cavity liners in procedures of selective caries removal from primary teeth, after 12 months of follow-up. The difficulty of this technique, however, is the subjective criterion to evaluate the amount of carious tissue that must be removed.^3^ Recently, it has been determined that the best guidance is the tactile sensation of the operator.^24^ In this study, the dentin consistency was taken into consideration. The selective caries removal was performed until firm dentin, resistant to cut, with a leather-like texture.^3^ ^,^ ^24^

Currently, the literature lacks consensus on the ideal liner to be used on the remaining affected dentin. CH is effective in reducing bacteria.^25^ MTA and PC are indicated for indirect pulp capping, but few authors studied their effects after selective caries removal.^11^ A survey found no statistically differences among CH, MTA, and PC after 6 months of follow-up.^11^ Other studies pointed out that the success of selective caries removal would not depend on the liner material,^17^ ^,^ ^26^ but on the hermetic sealing of the cavity. Some authors suggest that MTA and PC should be preferably used as liners.^11^ Thus, based on this lack of consensus randomized clinical trials should evaluate the strategies of caries removal and restorative procedures, including cavity liners.^22^

Some studies have used different *software* and techniques for measurements on scanned radiographs after procedures of indirect pulp capping in teeth.^18^ ^,^ ^20^ The size of the dentin barrier was determined by measuring the height of the pulp chamber^18^ on the immediate post-operative and control radiographs. The differences found between the obtained values resulted in the final measurements of the dentin barrier thickness. This measurement mechanism has some limitations; however, it was the method used to avoid mistaken measurements due to the difficulty in evaluating, especially in upper molar radiographs, the exact thickness of the dentin barrier. This fact occurred because the radioactivity of the material on the radiographic examination was masked by the overlapping of the radiographic image of the roots.

The literature points out that the evidence on the impact of selective caries removal on the restoration longevity is still scarce.^6^ The material indication should be guided by the carious lesion activity and the individual's specific conditions.^27^ ^,^ ^28^ Many materials are used to restore the primary teeth, but there is no consensus on the ideal material.^28^ The easiest clinical use of resin-modified glass ionomer cement, success in biological evaluation criteria and good performance {Croll, 2001, Clinical performance of resin-modified glass ionomer cement restorations in primary teeth. A retrospective evaluation}optimized its indication, with good outcomes in Class I, II, III and V cavities in primary teeth.^27^ ^–^ ^30^ As described in the literature and verified in the methodology of some studies,^11^ ^,^ ^20^ ^,^ ^22^ in this present study, the resin modified glass ionomer cement was the restorative material because of its good long-term success.

This study was the first randomized clinical trial that compared the clinical and radiographic outcomes of CH, MTA, and PC after the selective caries removal in primary teeth, however, the number of teeth used for analysis was small and more studies will be required. The occlusal caries that reach two-thirds of the dentin, without pulp involvement, was a difficult finding, since interproximal caries and deep cavities with pulp involvement were more commonly found in dental practice. The findings of this study are an additional step in the treatment of deep caries lesions through selective caries removal. Nevertheless, the long-term effects of therapy should be evaluated in future studies.

## Conclusion

The clinical and radiographic data showed that all cavity liners provided effective treatment of primary teeth after selective caries removal. The null hypothesis was accepted.
